# Complete genome sequence of a lumpy skin disease virus from a calf in the mid-northeastern region of Bangladesh

**DOI:** 10.1128/mra.00714-25

**Published:** 2025-10-07

**Authors:** Anandha Mozumder, Zakaria Al Noman, Roni Mia, S. M. Nazmul Hasan Siam, M. Shaminur Rahman, Mohammad Imtiaj Uddin Bhuiyan, M. Anwar Hossain, Sharmin Akter, Sukumar Saha, Tofazzal Islam, Md. Golzar Hossain

**Affiliations:** 1 Department of Microbiology and Hygiene, Bangladesh Agricultural University 54492, Mymensingh, Bangladesh; 2 Department of Microbiology, Jashore University of Science and Technology 421984, Jashore, Bangladesh; 3 Department of Environmental Health Science, University of Georgia 822554, Athens, Georgia, USA; 4 Department of Microbiology, University of Dhaka 95324, Dhaka, Bangladesh; 5 Department of Physiology, Bangladesh Agricultural University 54492, Mymensingh, Bangladesh; 6 Institute of Biotechnology and Genetic Engineering, Gazipur Agricultural University 198780, Gazipur, Bangladesh; Katholieke Universiteit Leuven, Leuven, Belgium

**Keywords:** lumpy skin disease virus, complete genome sequence, Bangladesh

## Abstract

Lumpy skin disease virus (LSDV) is currently causing significant mortality in young calves and presenting with distinct clinical features in Bangladesh. Here, we report the complete genome sequence of an LSDV strain from a calf in the mid-northeastern region of the country.

## ANNOUNCEMENT

Lumpy skin disease virus (LSDV) is a highly contagious pathogen responsible for lumpy skin disease, which affects cattle and water buffaloes of all ages. The virus is transmitted primarily through arthropod vectors and causes severe infections characterized by fever and multifocal cutaneous nodules (1). LSDV is a brick-shaped, enveloped double-stranded DNA virus belonging to the *Capripoxvirus* genus within the *Poxviridae* family. It measures approximately 293–299 nm in length and 262–273 nm in width, with a surface covered in tubular structures (2). The viral genome is approximately 151 kbp and encodes 156 proteins (3).

LSDV was first reported in Bangladesh in 2019 and has since become a significant burden on the livestock sector (4). Recently, distinct clinical and pathological features have been observed in affected cattle in Bangladesh (5). In this study, we performed whole-genome sequencing of the LSDV/Fulbaria-25-MGH-BD, obtained from a field outbreak in Fulbaria, Mymensingh, Bangladesh (24.6250° N, 90.2667° E).

A calf on the farm exhibited characteristic LSDV signs, including large nodules on various parts of the body. A sample from the necrotic skin nodule was collected using sterile surgical instruments and immediately transported to the laboratory under appropriate cold chain conditions. Viral inoculum was prepared by tissue homogenization following our previously published protocol and stored at −80°C (5). Viral DNA was extracted using the TIANamp Virus DNA/RNA Kit (Tiangen, China) per the manufacturer’s instructions. Next-generation sequencing was performed using the Illumina NovaSeq X Plus platform. Metagenomic libraries were prepared following the iNextEra protocol, including DNA tagmentation with Illumina bead-linked transposomes, adapter ligation, and limited-cycle PCR for indexing (6). The libraries were purified with AMPure XP beads, quantified using Qubit Flex, and sequenced to generate 2 × 150 bp paired-end reads at Novogene.

A total of 31 million reads were generated. Quality control of the FASTQ files was performed using FastQC (v0.11.6) (7). Adapter trimming and low-quality read removal were performed using Trimmomatic (v0.39) (8), employing a sliding window size of 4, a minimum average quality score of 20, and a minimum read length of 40 bp. Host DNA was depleted by aligning quality-filtered reads to the *Bos taurus* reference genome (ARS-UCD2.0) using BBMap (9), and the remaining unmapped reads were aligned to the LSDV reference genome (NC_003027; genome size: 150,773 bp) using BWA-MEM v0.7.17. Sorting and indexing were performed with SAMtools v1.12 (10), and the consensus genome was generated using BCFtools v1.12 and VCFtools v4.1 (11). The final consensus was derived from the *de novo* assembly of all host-filtered reads using Unicycler v 0.5.1, with the reference mapping serving as a validation guide to confirm genuine single-nucleotide polymorphisms (SNPs). While highly consistent with the reference-based mapping, which showed deep coverage (789×) with 856,749 reads mapped to the viral genome, covering more than 99.95% of the reference genome, minor differences were observed. These were resolved by manually inspecting each variant in a genome viewer, and genome annotation was completed with Prokka (v1.14.6) using default parameters (12). All tools were executed with default parameters in the Linux operating system, unless otherwise specified. The completeness of the assembled genome was confirmed by the presence of terminal repeat structures and raw read evidence spanning the contig ends. Genome annotation predicted 156 open reading frames (ORFs), encompassing the full complement of genes typical for an LSDV strain.

The final assembly resulted in a single contig of 150,723 nucleotides, representing a complete LSDV genome (LSDV/Fulbaria-25-MGH-BD). The assembled genome shares 99.90% and 99.89% identity with the reference genome (NC_003027) and a previously reported Bangladeshi strain (PP756497.1), respectively, as performed using the BLASTn. The GC content was 25.90%. This genome provides critical genetic information from a recent LSDV outbreak in northeastern Bangladesh and may contribute to the development of effective vaccines and control strategies.

## Data Availability

The complete genome sequence of LSDV/Fulbaria-25-MGH-BD has been deposited in GenBank, BioSample, and SRA under accession numbers PV022135, SAMN46412690, and PRJNA1215681, respectively.
